# Nurse understaffing associated with adverse outcomes for surgical admissions

**DOI:** 10.1093/bjs/znae215

**Published:** 2024-09-24

**Authors:** Paul Meredith, Lesley Turner, Christina Saville, Peter Griffiths

**Affiliations:** School of Health Sciences, University of Southampton, Southampton, UK; Research and Innovation, Portsmouth Hospitals University Trust, Portsmouth, UK; National Institute for Health Research Applied Research Collaboration Wessex, Southampton, UK; School of Health Sciences, University of Southampton, Southampton, UK; School of Health Sciences, University of Southampton, Southampton, UK; National Institute for Health Research Applied Research Collaboration Wessex, Southampton, UK; School of Health Sciences, University of Southampton, Southampton, UK; Research and Innovation, Portsmouth Hospitals University Trust, Portsmouth, UK; National Institute for Health Research Applied Research Collaboration Wessex, Southampton, UK

## Abstract

**Background:**

Nurses play a crucial role in maintaining the safety of surgical patients. Few nurse staffing studies have looked specifically at surgical patients to examine the impact of exposure to low staffing on patient outcomes.

**Methods:**

A longitudinal patient analysis was conducted in four organizations in England using routine data from 213 910 admissions to all surgical specialties. Patients’ staffing exposures were modelled as counts of understaffed registered nurse and nurse assistant days in the first 5 inpatient days. Understaffing was identified when staffing per patient-day was below the mean for the ward. Cox models were used to examine mortality within 30 days of admission and readmission within 30 days of discharge. Generalized linear models were used to investigate duration of hospital stay and occurrence of hospital-acquired conditions.

**Results:**

Increased exposure to registered nurse understaffing was associated with longer hospital stay and increased risk of deep vein thrombosis, pneumonia, and pressure ulcers. This was also true for nurse assistant understaffing, but the effect sizes tended to be smaller. In the Cox models, there were similarly increased hazards of death for registered nurse understaffing (HR 1.09, 95% c.i. 1.07 to 1.12) and nurse assistant understaffing (HR 1.10, 1.08 to 1.13), whereas the effect size of registered nurse understaffing for readmission (HR 1.02, 1.02 to 1.03) was greater than that seen with nurse assistants (HR 1.01, 1.01 to 1.02).

**Conclusion:**

Understaffing by both registered nurses and nursing assistants is associated with increased risks of a range of adverse events, and generally larger effects are seen with registered nurse understaffing.

## Teaser

This observational longitudinal study examined nurse staffing levels and their association with a variety of adverse patient outcomes for inpatient admissions to all surgical specialties. The patient-level data came from four disparate National Health Service hospital Trusts in England. The results indicated generally larger adverse effects for registered nurse understaffing compared with understaffing for nurse assistants (healthcare support workers and nurse associates).

## Introduction

It is estimated that one-third of the global burden of disease can be addressed by surgical care^1^ and over 300 million surgical procedures are carried out each year worldwide^2^. The Global Patient Safety Action Plan^3^ aims to achieve the maximum possible reduction in avoidable harms associated with healthcare. There are growing concerns about the quality of care received by adult patients undergoing surgery^4,5^, and the rising cost of avoidable complications, prolonged hospital stays, and readmissions^6,7^.

Patients in surgical units and ICUs are more likely to experience preventable harm than those in medical settings^8^. The safety of surgery has come under scrutiny, with never events and serious incidents being tracked as key measures of safety^9,10^. Many advances have been made in perioperative care, such as widespread use of the surgical safety checklist^11^, but preventable harms still occur. In a stratified meta-analysis^8^, the pooled prevalence of preventable harm was 10% for patients in surgical settings, and incidents relating to surgical procedures accounted for 23% of all harm incidents. It has been estimated that 55% of surgical-site infections are preventable^12^. Many safety interventions have focused on implementing checklists, staff training, and improving teamwork^13^. However, leaving the onus on frontline staff to prevent harms, without addressing shortfalls in nurse staffing levels, is a persistent barrier to patient safety^14^.

Variation in nurse staffing levels has been linked to patient safety consequences^15^. Hassen *et al.*^16^ conducted a Delphi study of international patient safety experts, who were asked to rank 17 factors contributing to care quality and safety on surgical wards. Nurse staffing was among the highest scoring factors in this consensus-generating activity. A systematic review^17^ including 44 studies found that higher nurse staffing levels were associated with lower 30-day mortality rates among surgical patients; however, 39 of these studies were cross-sectional in design using aggregated or sampled measures of nurse staffing^18–21^. Amiri *et al.*^22^ studied the relationship between nurse density and surgical complications in a panel analysis in 21 countries. They reported that an increase in nurse staffing levels was significantly associated with reductions in deep vein thrombosis (DVT), pulmonary embolism, postoperative sepsis, and wound dehiscence.

This was a multicentre longitudinal observational study in which daily variation in staffing was linked to individual patients to explore the relationship between nurse staffing levels and outcomes for surgical patients. Outcomes of interest were mortality, readmissions, duration of hospital stay, and the potentially avoidable clinical events of DVT, pneumonia, and pressure ulcers.

## Methods

This research comprises a subcohort analysis forming part of a study approved by the Health Research Authority (IRAS 273185) in England, with ethical review undertaken by the faculty research ethics committee at the University of Southampton (ERGO 52957).

Patient and staffing data sets were provided for the interval April 2015 to February 2020 by three hospital Trusts in the English National Health Service (NHS), and from April 2019 to Feb 2020 by the fourth. All data were supplied to researchers in pseudonymized form, with dates of birth replaced by 5-year age groups. The patient data were sourced from patient administration systems and the staffing data from electronic roster systems.

Inclusion criteria for admissions were that they: had a surgical treatment function code on admission as defined in the NHS data dictionary^23^, excluding children’s special services; included an overnight stay in an inpatient area, so patients having day surgery who did not stay overnight were excluded; included a stay in a unit other than maternity and paediatrics; and were for patients in the age group 15–19 years or above.

### Exposure, outcomes, and case-mix adjustment

Daily nursing staffing levels were obtained from records of worked shifts, which included temporary assignments to wards, but excluded absences, with details of the ward, pay band, and the shift fulfilment source (substantive contract, bank or agency) but no staff demographics. Nursing staff were categorized as registered nurse (RN) or nurse assistant (NA) according to pay band, with RNs having an NHS pay band of 5 or more. Daily ward staffing levels were calculated by job type (RN or NA) in hours per patient-day (HPPD) relative to the mean for the ward. Daily patient occupancy in a ward was calculated from patient movement records. Data cleaning resulted in the removal of less than 2% of ward day records from the data set (see the *Supplementary material* for details). Based on previous research^24^, exposures that occurred during the first 5 days of the hospital stay were used, accounting for most of the stay for most patients. A sensitivity analysis was undertaken using exposures occurring during the first 3 days and 10 days for comparison. In another sensitivity analysis, the models were adjusted for weekend admissions to test whether the differences in staffing of other occupational groups at weekends altered the effects of nurse understaffing.

Because a change in ward use would affect its staffing, a ward rebasing exercise was undertaken before calculating ward mean staffing levels. Where changes in a ward’s case mix were identified, the ward study interval was split into two at the change, with each subperiod given its own identifier. Each ward subperiod resulting from the split was then treated separately when calculating the expected staffing levels.

The outcomes were death from all causes within 30 days of admission, non-elective readmission within 30 days of discharge, duration of hospital stay, and the coding of three conditions that are not expected to be present in surgical admissions (pneumonia, DVT, and pressure ulcers). These three conditions were selected after considering nursing-sensitive outcomes from an umbrella review of research^25^ that could feasibly be identified in the available data. Death was determined either from a recorded discharge method of death or, for those discharged alive, from the date of death recorded posthumously on patient records via the national patient demographic service. Hence the outcome was not limited to in-hospital deaths. Readmission within 30 days of discharge was assigned to patients with a non-elective admission to the same Trust within 30 days of discharge. Duration of hospital stay was defined as the duration of continuous care in the Trust as an inpatient, and was calculated in hours from admission and discharge dates and times. Duration of stay for admissions that ended in discharge rather than death was modelled. Pressure ulcers, pneumonia, and DVT were identified from the ICD-10 diagnosis coding on the administrative record^26^.

To account for patient-level risk factors, a predicted risk of death using the published Summary Hospital Mortality Indicator (SHMI) model^27^ was calculated. This uses clinical and demographic factors (including age, method of admission, primary diagnosis, and co-morbidity) to calculate a predicted risk of death. These risk factors are also known to be predictive of readmission and duration of hospital stay. The SHMI risk coefficients from the April 2019 model (version 1.30) were used in the risk calculation.

### Statistical models

Admissions were regarded as being independent in the main analysis; however, a sensitivity analysis was carried out using only patient index admissions in the data set. Data set construction and all analyses were performed using R version 4.3.2 (R Core Team, Vienna, Austria); further details of the software and packages used can be found in the *Supplementary material*. *Table 1* summarizes the regression models used. Effect sizes were calculated as HRs, ORs, and linear multipliers along with 95% confidence intervals. There were no preplanned hypothesis tests.

**Table 1:** Regression models used for each study outcome

Outcome	Outcome type	Regression	Random effect	Effect type
All-cause mortality within 30 days of admission	Binomial	Cox proportional hazards mixed-effects regression with time-varying covariates	Ward for each patient-day	HR
Emergency readmission within 30 days of discharge	Binomial	Cox proportional hazards mixed-effects regression with fixed covariates	Discharge ward	HR
Duration of hospital stay	Days count	γ GLMM	Discharge ward	Linear multiplier
Deep vein thrombosis diagnosis	Binomial	Binomial GLMM	Ward after first night	OR
Pneumonia diagnosis	Binomial	Binomial GLMM	Ward after first night	OR
Pressure ulcer diagnosis	Binomial	Binomial GLMM	Ward after first night	OR

GLMM, generalized linear mixed model.

In mortality survival analysis, the hazard was modelled using patient-day observations and exposure to staffing measured by means of cumulative time-dependent covariates. This approach has been taken elsewhere^24,28^. Counts of understaffed days by job type were constructed with understaffing identified as a day where observed HPPD fell short of the expected (mean) HPPD. The model included the ward as a random effect to capture unspecified ward characteristics. If a patient-day included a ward transfer, the ward at the start of the day was used as the clustering ward. In the patient-period formatted survival analysis records, a previous day’s staffing values were carried forward in the event of missing values. The assumption of proportional hazards was examined using graphs of scaled Schoenfeld residuals and was seen to be reasonable for the staffing measures.

A Cox mixed-effects survival model was used to model time to readmission after discharge with fixed covariates. A patient’s exposure to staffing shortfall was calculated as the proportion of patient-days below the mean ward staffing level for each job type over the 5-day exposure period. The denominator count of exposure patient-days was adjusted for missing values. The time origin was the day of discharge and the discharge ward was included as a random effect. Graphs of scaled Schoenfeld residuals showed that the assumption of proportional hazards was reasonable for each predictor.

Duration of hospital stay is an outcome that exhibits a right-skewed distribution with mode near zero and heavy tails. Therefore the γ distribution was used in a generalized linear regression model using the same summary staffing measures as used in the readmissions model. The discharge ward was included as a random effect.

The ICD-10 diagnosis codes for the patient condition outcomes are listed in *Table S1*. The outcomes were identified from recorded ICD-10 codes for the hospital admissions but, because precise dates and times were not available, logistic regression rather than time-to-event models were used with a random effect for the ward occupied after the first night. As with the readmission and duration-of-stay analyses, proportions of understaffed days represented the staffing exposures with adjustment for missing values.

## Results

The most frequent admission specialties were trauma and orthopaedics, general surgery, and urology; these accounted for more than half of the admissions. Small admission numbers relating to oral, dentistry, burns, ophthalmic, and bariatric surgery were grouped into ‘other surgery’ for this description (*Table 2*). The 213 910 surgical admissions in the data set pertained to 162 598 patients, equivalent to 1.32 admissions per patient.

**Table 2:** Admission numbers by treatment function code

	No. of patients (*n* = 213 910)
Trauma and orthopaedics	54 608 (25.5)
General surgery	34 453 (16.1)
Urology	27 771 (13.0)
Ear nose and throat	14 906 (7.0)
Colorectal surgery	13 794 (6.4)
Neurosurgery	11 885 (5.6)
Plastic surgery	9430 (4.4)
Upper gastrointestinal surgery	9199 (4.3)
Spinal surgery	6386 (3.0)
Vascular surgery	5624 (2.6)
Hepatobiliary and pancreatic surgery	3961 (1.9)
Thoracic surgery	3850 (1.8)
Cardiac/cardiothoracic surgery	3573 (1.7)
Breast surgery	3494 (1.6)
Maxillofacial surgery	3465 (1.6)
Transplant surgery	2242 (1.0)
Other surgery*	5269 (2.5)

Values are *n* (%). *Oral, dentistry, burns, ophthalmic, and bariatric surgery.

The summary descriptives of the admissions overall, by death within 30 days of admission, and by emergency readmission within 30 days of discharge, are shown in *Table 3*. The modal 5-year age group was 70–74 years, 55.2% of admissions were as an emergency, female patients accounted for 49.2% of admissions, the median duration of hospital stay was 3.2 days, and 34.0% of the patients had a Charlson Co-morbidity Index score greater than 5^29^. The overall 30-day mortality rate was 2.1% and, among those discharged, the readmission rate within 30 days was 10.6%.

**Table 3:** Admissions cohort descriptives with mortality and readmission outcomes

	All (*n* = 213 910)	Alive (*n* = 209 378)	Died (*n* = 4532)	No readmission* (*n* = 187 791)	Readmitted (*n* = 22 309)
**Sex**					
Male	108 666 (50.8)	106 208 (50.7)	2458 (54.2)	94 870 (50.5)	11 768 (52.8)
Female	105 217 (49.2)	103 143 (49.3)	2074 (45.8)	92 895 (49.5)	10 540 (47.2)
Emergency admission	118 158 (55.2)	114 090 (54.5)	4068 (89.8)	99 633 (53.1)	15 072 (67.6)
**Age (years)**					
15–19	5277 (2.5)	5260 (2.5)	17 (0.4)	4902 (2.6)	360 (1.6)
20–24	8644 (4.0)	8628 (4.1)	16 (0.4)	7869 (4.2)	758 (3.4)
25–29	8752 (4.1)	8731 (4.2)	21 (0.5)	7844 (4.2)	890 (4.0)
30–34	9106 (4.3)	9088 (4.3)	18 (0.4)	8162 (4.3)	922 (4.1)
35–39	9241 (4.3)	9200 (4.4)	41 (0.9)	8228 (4.4)	980 (4.4)
40–44	9742 (4.6)	9677 (4.6)	65 (1.4)	8709 (4.6)	983 (4.4)
45–49	12 829 (6.0)	12 736 (6.1)	93 (2.1)	11 542 (6.1)	1208 (5.4)
50–54	15 709 (7.3)	15 566 (7.4)	143 (3.2)	14 013 (7.5)	1586 (7.1)
55–59	17 263 (8.1)	17 083 (8.2)	180 (4.0)	15 423 (8.2)	1693 (7.6)
60–64	17 739 (8.3)	17 484 (8.4)	255 (5.6)	15 832 (8.4)	1689 (7.6)
65–69	20 587 (9.6)	20 204 (9.6)	383 (8.5)	18 232 (9.7)	2023 (9.1)
70–74	23 245 (10.9)	22 672 (10.8)	573 (12.6)	20 378 (10.9)	2395 (10.7)
75–79	20 307 (9.5)	19 708 (9.4)	599 (13.2)	17 598 (9.4)	2207 (9.9)
80–84	17 336 (8.1)	16 588 (7.9)	748 (16.5)	14 602 (7.8)	2091 (9.4)
85–89	11 523 (5.4)	10 801 (5.2)	722 (15.9)	9318 (5.0)	1589 (7.1)
90–120	6610 (3.1)	5952 (2.8)	658 (14.5)	5139 (2.7)	935 (4.2)
**Charlson Co-morbidity Index score**					
0	104 771 (49.2)	104 734 (50.0)	601 (13.3)	95 997 (51.1)	8817 (39.5)
1–5	35 736 (16.7)	35 374 (16.9)	362 (8.0)	31 711 (16.9)	3726 (16.7)
> 5	72 396 (33.8)	68 833 (32.9)	3564 (78.6)	59 702 (31.8)	9708 (43.5)
Missing	442 (0.2)	437 (0.2)	5 (0.1)	381 (0.2)	58 (0.3)
% SHMI risk, mean (SD)	2.5 (5.8)	2.2 (5.2)	14.5 (13.4)	2.1 (5.0)	3.6 (6.8)
Duration of hospital stay (days), median (i.q.r.)	3.2 (1.5–7.1)	3.2 (1.5–7.0)	6.3 (2.7–12.2)	3.2 (1.5–6.6)	4.0 (1.9–9.1)

Values are *n* (%) unless otherwise indicated. *Discharged and not readmitted (excludes in-hospital deaths). SHMI, Summary Hospital Mortality Indicator.

Summary descriptive statistics for patient-days with regard to the hours of care received from RNs and NAs during the first 5 days of admissions are shown in *Table 4*. The distributions of care hours received were right-skewed such that 57% of patient-days were exposed to below mean levels for the ward.

**Table 4:** Staffing levels experienced during the first 5 days of patient stays

	Registered nurse staffing	Nurse assistant staffing
Hours per patient-day, mean(s.d.)	5.4(4.8)	2.8(1.4)
Hours per patient day, median (i.q.r.)	4.0 (3.1–5.6)	2.6 (1.9–3.4)
Days below mean staffing	465 653 (57.2)	466 408 (57.3)
Days at or above mean staffing	335 662 (41.2)	333 062 (40.9)
Days of unknown staffing	13 336 (1.6)	15 181 (1.9)

Values are *n* (%) unless otherwise indicated.

The results of the regression models showing the associations between below-mean staffing levels and patient outcomes are summarized in *Table 5*. The relative risk of death was increased by 9% (HR 1.09, 95% c.i. 1.07 to 1.12) with each day of low RN staffing and by 10% (HR 1.103, 1.08 to 1.13) with each day of low NA staffing. Although the readmissions model was also a survival analysis, the coefficients were not directly comparable because the staffing exposure variables were different and measured the proportion of low-staffed days rather than being the counts of low-staffed days. However, a 10% increase in the proportion of low-staff days can be interpreted as a change in the staffing level for one 12-h shift from above-mean staffing to below-mean staffing during a 5-day exposure period. Such an increase in RN understaffing increased the relative risk of readmission by 2% (HR 1.02, 1.02 to 1.03) and by 1% for NA staffing (HR 1.01, 1.01 to 1.02). The odds of the incidence of each of the three diagnosed conditions DVT, pneumonia, and pressure ulcers increased by 5, 6, and 6% respectively for each 10% increase in the proportion of days of low RN staffing, and correspondingly by 3, 5, and 3% respectively for each 10% increase in days of low NA staffing.

**Table 5:** Staffing levels and outcome associations from the adjusted models

	Effect size			Ward random effect (s.d.)
	Low registered nurse staffing	Low nurse assistant staffing	SHMI (risk)	
Mortality HR	1.09 (1.07, 1.12)	1.10 (1.08, 1.13)	1.08 (1.08, 1.09)	0.97
Readmission HR	1.02 (1.02, 1.03)	1.01 (1.01, 1.02)	1.02 (1.02, 1.02)	0.56
Duration of hospital stay multiplier	1.06 (1.06, 1.07)	1.05 (1.05, 1.06)	1.05 (1.05, 1.05)	0.97
Deep vein thrombosis OR	1.05 (1.02, 1.08)	1.03 (1.00, 1.06)	1.02 (1.01, 1.03)	0.76
Pneumonia OR	1.06 (1.05, 1.07)	1.05 (1.04, 1.06)	1.06 (1.06, 1.07)	1.02
Pressure ulcers OR	1.06 (1.04, 1.09)	1.03 (1.01, 1.05)	1.05 (1.05, 1.06)	0.88

Values in parentheses are 95% confidence intervals. SHMI, Summary Hospital Mortality Indicator.

The coefficients for the duration-of-stay model showed an increased length of 6% for each 10% change in the proportion of days of low RN staffing (coefficient 1.06, 95% c.i. 1.06 to 1.07) and a 5% increase for the same change in days of low NA staffing (coefficient 1.05, 1.05 to 1.05).

The sensitivity analysis using only patient index surgical admissions in the data set yielded effect estimates within the 95% confidence intervals of those shown in *Table 5* (*Table S2*). The results of sensitivity analysis comparing model coefficients for the low staffing predictors when the exposure was measured over different intervals—the first 3 days, 5 days, and 10 days of admission—are available in *Table S3*. The coefficients for the outcomes of mortality, readmission, and duration of hospital stay were similar for the three different exposure times. The effect sizes for three diagnosed patient conditions, however, became larger with an increase in measured exposure period. For a 10-day measured exposure, the odds of the incidence of each of DVT, pneumonia, and pressure ulcers increased by 6, 8, and 9% respectively for each 10% increase in the proportion of days of low RN staffing, and by 4, 6, and 5% correspondingly for changes in the proportion of days of low NA staffing. The sensitivity analysis that adjusted for weekend admissions showed very little change in coefficients for the low staffing variables, although the weekend admission co-variate was significant for mortality, duration of hospital stay, pneumonia, and pressure ulcers (*Table S4*).

## Discussion

In this longitudinal study of linked individual-patient data, the exposure to understaffing by either RNs or NAs was associated with a wide range of detrimental outcomes for patients admitted for surgery. The effects were larger for understaffing by RNs in most of the outcomes studied, but low NA staffing was still associated with increased risks. In the UK, RNs have graduate-level formal training and 2300 training hours of clinical placements before they can register with the professional council. In contrast NAs, commonly with the job title ‘healthcare support worker’, can be employed on probation with no formal training, but must complete a basic care certificate which includes competencies in fluids and nutrition, infection prevention and control, and basic life support^30^. Only registered staff create care plans, and administer medications, infusions, and other treatments. NAs work under the guidance of RNs and support them in the delivery of nursing services. It is likely that NA understaffing results in an increased workload for RNs, which may in turn mean that some care tasks are delayed or left undone.

The cost of readmissions and avoidable events is significant. Increased duration of hospital stay is the primary cost associated with healthcare-acquired infection^31^, and adult inpatient healthcare-acquired infections in general and teaching hospitals were estimated in 2016–2017 to cost NHS England £2.1 billion annually^32^. Treatment costs for morbidity, and the potential reduction in duration and quality of life would be added to this burden. This is one of the first studies that has used a longitudinal design for exploring nurse staffing levels and a large range of outcomes in surgical patients.

Previous studies^33–36^ of surgical populations also identified associations between RN understaffing and mortality. When the subset of surgical patients was examined in the RN4CAST study^37^, it was found that increasing a nurse’s workload by one patient was associated with a 16% increased odds of death. However, in RN4CAST, in common with many other studies, the staffing levels were based on estimates of hospital-level averages over long periods, and did not specifically estimate staffing on surgical units. The present study measured staffing at a more granular level using ward rosters, and individual patients were followed up longitudinally. The strength of this approach is that exposures were calculated more accurately, patients were followed over time, and more detailed case-mix adjustment was undertaken.

A longitudinal study^24^ also found that the hazard of death was increased for every day a patient was exposed to understaffing by RNs in a mixed medical and surgical population. The picture for NAs is less clear in the literature. Most studies^36,38,39^ suggested that there is a hazardous effect of higher NA staffing, but one^24^ reported a non-linear relationship between the hazard of death and differing NA levels, with harm being associated with both low and high levels.

The observed associations between higher staffing and reduced mortality and morbidity are important. There has been limited progress in improving patient safety outcomes despite intense interest in this area. High-profile campaigns, cultural shifts, training, and interventions have not yet offered significant and sustainable benefits^14^. A recent review suggested that investment in RNs can be highly cost-effective^40^, although more evidence is needed. The present findings add to the evidence that staffing levels merit more attention.

The method of measuring exposure for the first 5 days of each admission did not identify all associations between understaffing and adverse events that occurred later in admissions because the understaffing may have occurred after the 5 days. This might have been the case with hospital-acquired conditions such as pressure ulcers, pneumonia, and DVT, as demonstrated by the results in *Table S3*, which showed larger effects for understaffing with longer measured exposure periods. Overall, the 5-day exposure period was effective in finding variations in staffing which could be associated with subsequent adverse events. Longer exposure periods have less daily variation in staffing levels. There is scope for methods research to identify measures of understaffing that best associate the staffing exposure with the outcome for different kinds of adverse event in longitudinal studies. A limitation of the data set was that there was no visibility of hospital-acquired conditions manifesting themselves after discharge unless they resulted in readmission.

Although this study demonstrated an association between nurse understaffing and negative outcomes, the authors are unable to recommend an optimal level of staffing based on the findings. There are further unanswered questions because data on medical staff or other support staff such as physiotherapists were not accessible. Higher staffing levels across the wider multidisciplinary team, including medical and allied health professional staff, have also been associated with improved outcomes^41^. However, the effects of nurse understaffing in the present models would be altered only if there were a correlation between nurse understaffing and understaffing in the other staff groups. The sensitivity analysis with weekend admissions as a covariate showed little change in the nurse understaffing coefficients, suggesting that nurse understaffing is largely independent of understaffing in other occupational groups.

This study was conducted in four acute NHS Trusts in England, and the sample was heterogeneous, representing a range of catchment areas, socioeconomic groups, patient demographics, population health indicators, Trust sizes, and services. However, it is an observational study without matching controls and the data related to a number of years, so it is possible that there were organizational changes and service improvements during the period that might have affected patient safety.

## Supplementary Material

znae215_Supplementary_Data

## Data Availability

The nature of the data (individual patient data) and the data sharing agreements with the data providers means we are unable to share data.
