# Corrigendum to: Nurse understaffing associated with adverse outcomes for surgical admissions

**DOI:** 10.1093/bjs/znae267

**Published:** 2024-10-15

This is a corrigendum to: Paul Meredith, Lesley Turner, Christina Saville, Peter Griffiths, Nurse understaffing associated with adverse outcomes for surgical admissions, *British Journal of Surgery*, Volume 111, Issue 9, September 2024, znae215, https://doi.org/10.1093/bjs/znae215

In the originally published version of this manuscript, an error was present in the following sentence, whereby “3, 53, and 3%” should have been given as “3, 5, and 3%”:

The odds of the incidence of each of the three diagnosed conditions DVT, pneumonia, and pressure ulcers increased by 5, 6, and 6% respectively for each 10% increase in the proportion of days of low RN staffing, and correspondingly by 3, 53, and 3% respectively for each 10% increase in days of low NA staffing.

The sentence has been corrected to read as follows:

The odds of the incidence of each of the three diagnosed conditions DVT, pneumonia, and pressure ulcers increased by 5, 6, and 6% respectively for each 10% increase in the proportion of days of low RN staffing, and correspondingly by 3, 5, and 3% respectively for each 10% increase in days of low NA staffing.

