# Supplementary material for: Nurse understaffing associated with adverse outcomes for surgical admissions
Source: Br J Surg. 2024 Sep 24;111(9):znae215. doi: 10.1093/bjs/znae215 (PMC11419734; doi:10.1093/bjs/znae215)
Supplement: znae215_Supplementary_Data [file znae215_supplementary_data.docx]

Associations between adverse outcomes for surgical admissions and nurse understaffing – a longitudinal study

Paul Meredith^1,2,3^

Lesley Turner^1^

Christina Saville^1,3^

Peter Griffiths^1,2,3^

^1^ School of Health Sciences, University of Southampton, Southampton, UK

^2^ Research and Innovation, Portsmouth Hospitals University Trust, Portsmouth, UK

^3^ Applied Research Collaboration Wessex, University of Southampton, Southampton, UK

**Corresponding author.** Dr Paul Meredith, Senior Research Fellow, School of Health Sciences, University of Southampton, Highfield Campus, Southampton, SO17 1BJ, United Kingdom **ORCID ID** 0000-0002-5464-371X

**Supplementary Materials - Index**

| **Supplementary Methods** |  |
| --- | --- |
| Data cleaning | *page 2* |
| Statistical software | *page 3* |
| **Supplementary Figures and Tables** |  |
| Table S1: ICD-10 Codes identifying the patient condition outcomes | *page 4* |
| Table S2: Staffing levels and outcome associations from the adjusted models for patient index admissions | *page 5* |
| Table S3: Comparison of model staffing coefficients for different periods of measured staffing | *page 6* |
| Table S4: Staffing levels and outcome associations from the adjusted models with weekend admission predictor | *page 7* |
|  |  |

**Supplementary Methods**

**Data Cleaning**

Unrealistic staffing levels on patient days were replaced by missing values in the dataset. Likewise any gaps in staffing records after matching to ward days were filled with missing values. This amounted to around 1.64% of patient days with missing RN staffing levels and 1.86% days with missing NA levels.

When analysing length of stay, small numbers of admissions with a length of stay of 180 days or more were trimmed from the dataset. Occurrences of this were more likely to indicate a late closure of the admission record than a genuine stay exceeding that interval.

**Software**

All analyses were performed using R Statistical Software (v4.3.2)^1^ in the RStudio integrated development environment (v2023.12.1.402)^2^. The tidyverse R package (v2.0.0)^3^ was used for data wrangling. The survival analysis data set used to model mortality and readmission was constructed with the survival R package (v3.5.8)^4^ and analysed with the coxme R package (v2.2.18.1)^5^. The length of stay gamma regression modelling and patient conditions logistic modelling were undertaken with the lme4 R package (V1.1.35.1)^6^. Descriptive statistics used the finalfit R package (v1.0.7)^7^. The rmarkdown R package (v2.25)^8^ was used to format output in the preparation of this paper.

1 R Core Team (2023). _R: A Language and Environment for Statistical Computing_. R Foundation for Statistical Computing, Vienna, Austria. <https://www.R-project.org/>.

2 Posit team (2024). RStudio: Integrated Development Environment for R. Posit Software, PBC, Boston, MA. URL <http://www.posit.co/>.

3 Wickham H, Averick M, Bryan J, Chang W, McGowan LD, François R, Grolemund G, Hayes A, Henry L, Hester J, Kuhn M, Pedersen TL, Miller E, Bache SM, Müller K, Ooms J, Robinson D, Seidel DP, Spinu V, Takahashi K, Vaughan D, Wilke C, Woo K, Yutani H (2019). “Welcome to the tidyverse.” _Journal of Open Source Software_, *4*(43), 1686. doi:10.21105/joss.01686 <https://doi.org/10.21105/joss.01686>

4 Terry M. Therneau, Patricia M. Grambsch (2000). _Modeling Survival Data: Extending the Cox Model_. Springer, New York. ISBN 0-387-98784-3.

5 Therneau TM (2022). _coxme: Mixed Effects Cox Models_. R package version 2.2-18.1, <https://CRAN.R-project.org/package=coxme>.

6 Douglas Bates, Martin Maechler, Ben Bolker, Steve Walker (2015). Fitting Linear Mixed-Effects Models Using lme4. Journal of Statistical Software, 67(1), 1-48. doi:10.18637/jss.v067.i01.

7 Harrison E, Drake T, Pius R (2023). _finalfit: Quickly Create Elegant Regression Results Tables and Plots when Modelling_. R package version 1.0.7, <https://CRAN.R-project.org/package=finalfit>.

8 Allaire J, Xie Y, Dervieux C, McPherson J, Luraschi J, Ushey K, Atkins A, Wickham H, Cheng J, Chang W, Iannone R (2023). _rmarkdown: Dynamic Documents for R_. R package version 2.25, <https://github.com/rstudio/rmarkdown>.

**Supplementary Figures and Tables**

Table S1: ICD-10 Codes identifying the patient condition outcomes

| **Patient outcome** | **ICD-10 Code and Description** |
| --- | --- |
| Pneumonia | **Diagnosis codes** "J13 Pneumonia due to Streptococcus pneumoniae","J14 Pneumonia due to Haemophilus influenzae","J15 Bacterial pneumonia, not elsewhere classified","J16 Pneumonia due to other infectious organisms, not elsewhere classified","J18 Pneumonia, organism unspecified". |
| Deep venous thrombosis | **Diagnosis codes** “I80.1 Phlebitis and thrombophlebitis of femoral vein”, “I80.2 Phlebitis and thrombophlebitis of other deep vessels of lower extremities”, “I80.3 Phlebitis and thrombophlebitis of lower extremities, unspecified”.  Matthews, A and Bhaskaran, K (2018). Clinical code list - Deep Vein Thrombosis. [Data Collection]. London School of Hygiene & Tropical Medicine, London, United Kingdom. <https://doi.org/10.17037/DATA.00000733> |
| Pressure ulcer | **Diagnosis codes** “L89.2 Stage III decubitus ulcer”, “L89.3 Stage IV decubitus ulcer”, “L89.9 Decubitus ulcer and pressure area, unspecified ie without mention of stage” |

Table S2: Staffing levels and outcome associations from the adjusted models for patient index admissions

| **Outcome** | **Low RN** | **Low NA** | **SHMI (Risk)** | **Ward random effect (SD)** |
| --- | --- | --- | --- | --- |
| **Mortality** | 1.088 (1.059-1.119) | 1.092 (1.062-1.122) | 1.085 (1.083-1.088) | 1.012 |
| **Readmission** | 1.023 (1.017-1.029) | 1.019 (1.013-1.025) | 1.020 (1.018-1.022) | 0.540 |
| **Length of stay** | 1.062 (1.059-1.064) | 1.055 (1.053-1.058) | 1.057 (1.055-1.058) | 1.356 |
| **Deep-vein thrombosis** | 1.065 (1.032-1.099) | 1.026 (0.996-1.058) | 1.011 (0.999-1.024) | 0.749 |
| **Pneumonia** | 1.056 (1.046-1.067) | 1.053 (1.043-1.063) | 1.065 (1.062-1.068) | 1.051 |
| **Pressure ulcers** | 1.070 (1.044-1.096) | 1.041 (1.017-1.065) | 1.054 (1.048-1.061) | 0.867 |

95% confidence interval limits given in brackets;

Table S3:. Comparison of model staffing coefficients for different periods of measured staffing

|  |  | **Measured exposure period** | | |
| --- | --- | --- | --- | --- |
| **Outcome** | **Staffing predictor** | **3 days** | **5 days** | **10 days** |
| **Mortality** | **Low RN** | 1.095 (1.057-1.135) | 1.092 (1.066-1.118) | 1.083 (1.067-1.100) |
|  | **Low NA** | 1.121 (1.082-1.162) | 1.103 (1.077-1.129) | 1.080 (1.064-1.096) |
| **Readmission** | **Low RN** | 1.023 (1.018-1.028) | 1.023 (1.019-1.028) | 1.026 (1.021-1.031) |
|  | **Low NA** | 1.015 (1.010-1.019) | 1.014 (1.010-1.019) | 1.015 (1.010-1.020) |
| **Length of Stay** | **Low RN** | 1.063 (1.061-1.065) | 1.063 (1.061-1.065) | 1.076 (1.074-1.079) |
|  | **Low NA** | 1.055 (1.053-1.057) | 1.054 (1.052-1.057) | 1.064 (1.062-1.066) |
| **Deep-vein thrombosis** | **Low RN** | 1.031 (1.006-1.056) | 1.048 (1.020-1.077) | 1.062 (1.032-1.093) |
|  | **Low NA** | 1.024 (1.000-1.049) | 1.029 (1.002-1.056) | 1.041 (1.012-1.070) |
| **Pneumonia** | **Low RN** | 1.029 (1.021-1.037) | 1.057 (1.048-1.066) | 1.077 (1.067-1.087) |
|  | **Low NA** | 1.028 (1.020-1.035) | 1.051 (1.042-1.059) | 1.065 (1.056-1.074) |
| **Pressure Ulcer** | **Low RN** | 1.034 (1.015-1.052) | 1.064 (1.043-1.085) | 1.090 (1.067-1.114) |
|  | **Low NA** | 1.013 (0.995-1.031) | 1.029 (1.009-1.049) | 1.045 (1.024-1.067) |

95% confidence interval limits given in brackets;

Table S4: Staffing levels and outcome associations from the adjusted models with weekend admission predictor

| **Outcome** | **Low RN** | **Low NA** | **Wke_adm^*^** | **SHMI (Risk)** | **Ward random effect (SD)** |
| --- | --- | --- | --- | --- | --- |
| **Mortality** | 1.091 (1.065-1.118) | 1.101 (1.075-1.127) | 1.247 (1.165-1.335) | 1.084 (1.082-1.086) | 1.012 |
| **Readmission** | 1.023 (1.019-1.028) | 1.014 (1.010-1.019) | 1.021 (0.986-1.058) | 1.018 (1.016-1.020) | 0.540 |
| **Length of stay** | 1.063 (1.061-1.065) | 1.054 (1.052-1.057) | 1.081 (1.063-1.099) | 1.053 (1.052-1.054) | 1.356 |
| **Deep-vein thrombosis** | 1.048 (1.020-1.076) | 1.028 (1.002-1.056) | 1.083 (0.883-1.328) | 1.016 (1.006-1.026) | 0.749 |
| **Pneumonia** | 1.057 (1.048-1.066) | 1.051 (1.042-1.059) | 1.141 (1.072-1.215) | 1.065 (1.062-1.067) | 1.051 |
| **Pressure ulcers** | 1.064 (1.043-1.085) | 1.029 (1.009-1.049) | 1.165 (1.008-1.346) | 1.052 (1.046-1.057) | 0.867 |

* Weekend admission: hospital admission on Saturday or Sunday
